# Isoprene-degrading bacteria associated with the phyllosphere of *Salix fragilis,* a high isoprene-emitting willow of the Northern Hemisphere

**DOI:** 10.1186/s40793-021-00386-x

**Published:** 2021-08-26

**Authors:** Lisa Gibson, Andrew T. Crombie, Niall P. McNamara, J. Colin Murrell

**Affiliations:** 1 School of Environmental Sciences, University of East Anglia, Norwich Research Park, Norwich, NR4 7TJ UK; 2 School of Biological Sciences, University of East Anglia, Norwich Research Park, Norwich, NR4 7TJ UK; 3 Centre of Ecology and Hydrology, Lancaster University, Bailrigg, Lancaster, LA1 4AP UK

**Keywords:** Isoprene, Climate, Isoprene monooxygenase, DNA stable isotope probing, *Salix fragilis*, Willow tree, *isoA*

## Abstract

**Background:**

Isoprene accounts for about half of total biogenic volatile organic compound emissions globally, and as a climate active gas it plays a significant and varied role in atmospheric chemistry. Terrestrial plants are the largest source of isoprene, with willow (*Salix*) making up one of the most active groups of isoprene producing trees. Bacteria act as a biological sink for isoprene and those bacteria associated with high isoprene-emitting trees may provide further insight into its biodegradation.

**Results:**

A DNA-SIP experiment incubating willow (*Salix fragilis*) leaves with ^13^C-labelled isoprene revealed an abundance of *Comamonadaceae, Methylobacterium, Mycobacterium* and *Polaromonas* in the isoprene degrading community when analysed by 16S rRNA gene amplicon sequencing. Metagenomic analysis of ^13^C-enriched samples confirmed the abundance of *Comamonadaceae, Acidovorax, Polaromonas, Variovorax* and *Ramlibacter. Mycobacterium* and *Methylobacterium* were also identified after metagenomic analysis and a *Mycobacterium* metagenome-assembled genome (MAG) was recovered. This contained two complete isoprene degradation metabolic gene clusters, along with a propane monooxygenase gene cluster. Analysis of the abundance of the alpha subunit of the isoprene monooxygenase, *isoA,* in unenriched DNA samples revealed that isoprene degraders associated with willow leaves are abundant, making up nearly 0.2% of the natural bacterial community.

**Conclusions:**

Analysis of the isoprene degrading community associated with willow leaves using DNA-SIP and focused metagenomics techniques enabled recovery of the genome of an active isoprene-degrading *Mycobacterium* species and provided valuable insight into bacteria involved in degradation of isoprene on the leaves of a key species of isoprene-emitting tree in the northern hemisphere.

**Supplementary Information:**

The online version contains supplementary material available at 10.1186/s40793-021-00386-x.

## Introduction

Isoprene (2-methyl-1,3-butadiene) is a biogenic volatile organic compound (BVOC) that is emitted globally at a rate of ~ 500 Tg year^−1^ [1] making isoprene one of the most prevalent atmospheric BVOCs, second only to methane [1, 2]. Due to its volatile nature as a reactive diene, isoprene plays a complex role in atmospheric chemistry and is thought to contribute both warming and cooling effects on the Earth’s climate. Isoprene reacts readily with hydroxyl radicals (OH), reducing the oxidative capacity of the atmosphere and increasing the residence time of other greenhouse gases like methane. In pristine environments with low levels of nitrogen oxides (NO_x_), isoprene reacts directly with OH, thus reducing its overall tropospheric level. However in the presence of higher NOx, common in highly populated urban areas, the oxidation of isoprene results in the production of NO_2_ via photolysis, which in turn increases ozone levels and thus can have a detrimental impact on air quality and human health [3, 4]. In other circumstances, the products of isoprene oxidation in the atmosphere can act as cloud condensation nuclei which then lead to an increase in cloud formation and contribute to atmospheric cooling [5].

The production of cis-polyisoprene (synthetic rubber) is the main source of anthropogenic emissions of isoprene [6] but the bulk of isoprene produced arises from the natural environment. Biosynthesis of isoprene is widespread and can be observed in some species of bacteria, fungi, algae and animal in both aquatic and terrestrial environments [7–14], however, about 90% of isoprene production originates from terrestrial plants and particularly trees [12, 15]. Isoprene synthase is the enzyme responsible for isoprene production in plants, although its presence and activity can vary significantly, even between trees of the same genus [16–19]. In trees that emit isoprene, it is produced in the chloroplast via the methyl-erythritol 4-phosphate (MEP) pathway [20]. The isoprene synthase enzyme is responsible for converting dimethylallyl diphosphate (DMADP) to isoprene. In some cases, 1–2% of the total carbon fixed by the plant is converted to isoprene, making its production a significant investment on the part of the plant [21, 22]. However, the exact reason why plants produce isoprene is not yet fully understood. It has been reported that isoprene improves the resilience of plants to oxidative, thermal and biotic stresses [22–25], however the mechanisms for these processes have yet to be fully elucidated. In terms of thermo-tolerance, it was previously thought that isoprene could intercalate into thylakoid membranes and improve their stability under heat stress [26, 27]. However, recent studies show that due to the highly volatile nature of isoprene and its inability to dissolve well into cellular components, isoprene is unable to accumulate in chloroplastic membranes at a concentration high enough to provide any significant impact on membrane stability [28]. A review by Lantz et al. [29] suggests that isoprene may play a role in gene expression, although direct evidence for this theory is still limited.

The production and impact of isoprene on the atmosphere has long been studied, but the removal of isoprene via biological processes is a mechanism that is still relatively unexplored. Field chamber studies showed that temperate forest soils can rapidly deplete isoprene from ~ 400 ppbv to below a 5 ppbv detection limit [30, 31]. Experiments utilising a continuous-flow method showed that temperate forest soil systems were also effective at consuming lower concentrations of isoprene, with as little as 2 ppbv isoprene being utilised [32]. Bacterial strains capable of growth with isoprene as their sole carbon source have been isolated from soil, phyllosphere, and aquatic environments [33–40], reviewed in [41]. These earlier studies found most success in isolating Gram-positive Actinobacteria such as *Rhodococcus, Gordonia* and *Mycobacterium.* Recently however, targeted isolation techniques have resulted in the isolation of novel Gram-negative Proteobacteria such as *Sphingopyxis*, *Variovorax* and *Ramilibacter*, diversifying the collection of validated isoprene degrading bacteria [42].

All extant isoprene degrading bacteria use the enzyme isoprene monooxygenase (IsoMO) to oxidise isoprene. IsoMO, encoded by the genes, *isoABCDEF*, catalyses the first step of the isoprene degradation pathway. Adjacent genes *isoGHIJ* encode a CoA transferase, dehydrogenase and two glutathione transferases, and glutathione biosynthesis genes and other putative genes involved in the subsequent steps of isoprene metabolism (recently reviewed in [41, 43] are also in the same cluster. The gene encoding the α-subunit of the IsoMO, *isoA,* is highly conserved amongst isoprene degrading bacteria, making it an excellent functional gene probe when investigating the presence, distribution, and diversity of isoprene degrading bacteria in the environment. This approach (recently reviewed in [44]) has been utilised previously in combination with DNA stable isotope probing (DNA-SIP) [45, 46] to investigate isoprene degrading communities in a cultivation-independent manner [36, 37, 39–42, 47]. The genetic information recovered, and the isolates obtained from these studies allowed for the development of new, robust gene probes to examine the diversity of isoprene degradation genes recovered from environmental samples [48].

Willow species are common in the northern hemisphere and are among the highest emitters of isoprene (emissions of up to 37 µg g(dry weight) h^−1^ have been recorded) [49]. There are a number of willow species in the UK such as the *Salix fragilis* studied here, one of the larger species of Willow often found by rivers and lakes and frequently used to stabilise riverside soil [50]. Willow is also planted in high numbers as a short-rotation coppice (SRC) used for bioenergy, an example of which was also examined in this study [51–53].

Soil associated with willow species has previously been investigated for the presence of isoprene degrading bacteria [42] however, at 30 ppmv, the level of isoprene found in the intercellular spaces of leaves is orders of magnitude higher than atmospheric isoprene found at ground level, with the potential to select for a very different isoprene-degrading bacterial community than that found in bulk soil environments [54, 55]*.* The aim of this study was to investigate the phyllosphere of willow using DNA-SIP and qPCR methods to identify the bacteria responsible for isoprene degradation on the leaves of a high-isoprene-emitting tree in the Northern Hemisphere.

## Materials and methods

### Isoprene DNA-SIP incubations and DNA extraction

For SIP incubations, leaves (approximately 2.5 m above ground level) were removed from the south-facing side of a willow tree (*Salix fragilis*), located on the campus of the University of East Anglia. Cells were dislodged from leaves (approx. 5 g) by ultrasound as described previously [40] except using a 1/2 dilution of the minimal medium [56]. Cell pellets (retrieved by centrifugation and filtration as described [40]) were resuspended in 50 ml minimal medium diluted as above and incubated in flasks (2 L volume) with isoprene (either unlabelled (Sigma Aldrich, Gillingham, UK) or uniformly ^13^C-labelled, synthesised as described [39]) added to approx. 150 ppmv by injection of vapour through the septum and incubated with shaking (150 rpm) at 25 °C. Headspace isoprene concentrations were monitored by gas chromatography [37] and incubations with labelled or unlabelled substrate were carried out in triplicate. When isoprene was depleted, flasks were replenished once to the same isoprene concentration, and cells were harvested when the microcosms had consumed approx. 0.5 µmol isoprene ml^−1^ (13–53 days). Cells were harvested by centrifugation (12,000× *g*, 20 min, 15 °C) and the cell pellet stored at − 20 °C prior to DNA extraction.

### Nucleic acid extraction

DNA was extracted using the FastDNA spin kit for soil (MP Biomedicals, Solon, OH, USA) following the manufacturer’s instructions, except using two bead beating treatments (each 40 s, speed 6.0) in the FastPrep instrument. DNA was quantified using a Qubit 2.0 fluorometer (Thermo Fisher, Waltham, MA, USA) following the manufacturer’s instructions.

### Stable isotope probing

^13^C-labelled and unlabelled DNA were separated by density gradient ultracentrifugation and fractionation (12 fractions per sample) as described previously [39]. The relative proportion of DNA retrieved from each fraction was plotted against buoyant density, quantified by refractometry (Reichert AR200, Reichert Analytical Instruments, Buffalo, NY, USA), (Additional file 1: Fig. S1). Based on the data shown in Additional file 1: Fig. S1 the fractions containing ^13^C-labelled (“heavy”) and unlabelled (“light”) DNA were identified and used for analysis. Of the total DNA recovered from each ultracentrifugation tube, on average 1.2 ± 0.47% was located in the heavy fractions of ^12^C-isoprene incubations, whereas 19.7 ± 2.53% was recovered from the heavy fractions of ^13^C incubations.

### Sequencing of DNA

The bacterial communities of the timepoint zero samples together with the heavy and light fractions from ^12^C- and ^13^C-isoprene incubations were profiled by amplicon sequencing of the 16S rRNA gene, generated using primers 0341F/0785R [57], following the Illumina 16S Metagenomic Sequencing Library Preparation (2013) protocol [58]. Pooled libraries were sequenced using the Illumina MiSeq platform (2 × 250 bp paired-end reads) at the Centre for Genomic Research (CGR), University of Liverpool, UK. DNA from the heavy fractions of ^13^C-isoprene incubations was also sequenced by shotgun metagenomics. Library preparation (insert size < 500 bp) and sequencing were conducted by CGR using an Illumina HiSeq 2500 platform in high-output mode (v4) (2 × 125 bp paired-end reads). Samples were processed with the use of the Nextera XT kit following the Nextera XT workflow [59] including an additional purification step with the use of Agencourt AMPure XP beads.

### 16S rRNA gene amplicon sequencing

16S rRNA gene amplicon sequencing data were analysed using the Bioconductor package DADA2 ([60]; version 1.6). Forward and reverse reads were trimmed by 33 and 37 nucleotides respectively to remove any adapter sequences and quality-filtered if their expected error was greater than two according to the DADA2 quality analysis. Sequences were then denoised using the estimated error rates and resultant reads were dereplicated. Subsequently, chimeric sequences were discarded and the DADA2 algorithm was used to infer individual amplicon sequence variants (ASVs). ASVs were then taxonomically identified with the use of the RDP rRNA database ([61]; version RDP trainset 18).

### Metagenomic analysis

ofThe phylogenetic community as derived by raw metagenomic reads was assessed via Kraken ([62]; version 1.1.1) and the results fed to Bracken (version 2.5; [63]) with a kmer length of 31 to determine relative abundance of each taxa. Metagenomic reads were processed and initial analysis carried out with various modules included in the MetaWRAP pipeline as described here ([64]; version 1.2.1). Sequencing results from the heavy fractions of the three ^13^C-isoprene enriched samples were pooled giving a total of 24,849,791 reads and the subsequent assembly of these reads resulted in an N50 of 15,027 bp. To achieve this, raw, pooled reads were pre-processed with the use of the metaWRAP::Read_qc module with default settings, though the bmtagger step was skipped. Assembly was carried out with the metaWRAP::Assembly module utilising the assembler metaSPAdes.

Binning of metagenome assembled genomes (MAGs) was carried out simultaneously via the metaWRAP::Binning module using metaBAT2 ([65]; version 2.12.1), MaxBin2 ([66]; version 2.2.6) and CONCOCT ([67]; version 1.0.0) and the results for each compared in order to compile the MAGs of the highest quality from each. These MAGs were then reassembled to improve completion by mapping reads back to the assembled genomes. The completeness, strain heterogeneity and contamination of each MAG was assessed with CheckM (version 1.0.18; [68]) utilising the lineage-specific workflow. The metaWRAP::Classify bins module [63] was used to assign taxonomy to each MAG. MAGs of interest were functionally annotated with PROKKA ([69]; version 1.14.5) with default settings and the ‘–centre X’ tag to generate appropriate contig names. MAG abundance was measured as genome copies per million reads. This measure was obtained by aligning reads to the entire indexed metagenomic assembly using the Quant_bins::MetaWRAP module which then uses Salmon ([70]; version 0.14.2) to estimate the abundance of each contig. The average abundance of each MAG is calculated by taking the length-weighted average of the MAG’s contig abundance [71]. Annotated MAGs were investigated with the use of the Artemis genome browser [72]. MAGs were investigated for the presence of plasmid DNA with the use of the plasmidVerify script [73] developed by the Centre for Algorithmic Biotechnology, Saint Petersburg State University.

MAGs of interest were further analysed with the MiGA pipeline to obtain a higher resolution taxonomic classification to species level where possible [74].

### *isoA* qPCR analysis

For qPCR analysis of isoprene degraders in the environment, samples were obtained from a commercial farm in Lincolnshire, NE England (53°18′55″N; 0°34′40″W) on adjacent plantations of willow SRC (*Salix viminalis*), miscanthus (*Miscanthus x giganteus*) and poplar (*Populus nigra*). Three different trees each made up the replicates for poplar and willow samples. From each of these replicates, three samples per tree were made up of 2 g of collected leaf material taken from different sides of each tree. *Miscanthus* leaves were taken from six varying locations within the plantation area. DNA was extracted from cells associated with leaves as described earlier for DNA-SIP experiments. Extracted DNA was cleaned of any impurities that may have inhibited PCR activity with a second run of the FastDNA spin kit for soil (MP Biomedicals, Solon, OH, USA) following the manufacturer’s instructions. DNA was quantified as described earlier.

*isoA* sequence abundance in unenriched environmental samples were quantified by qPCR targeting the *isoA* gene using primers isoA14F and isoA511R [48]. qPCR assays were carried out with a StepOne Plus real-time PCR instrument (Applied Biosystems, Waltham, MA, USA). qPCR reactions (20 μl) contained 1–18 ng of DNA, 400 nM of each primer and 10 μl of SensiFast SYBR Hi-ROX kit (Bioline, Memphis, TN, USA). The qPCR reaction consisted of an initial denaturation step at 95 °C for 3 min, followed by 40 cycles of 95 °C for 20 s, 60 °C for 20 s and 72 °C for 30 s. Data were acquired at 88 °C for 15 s to avoid quantification of primer dimers. Agarose gel electrophoresis and melting curves obtained by increasing the temperature in 0.3 °C increments from 60 to 95 °C were used to determine specificity of qPCR reactions. *isoA* gene copy numbers were determined from qPCR of ten-fold dilution series with DNA standards. Standards were prepared by cloning the *isoA* gene of *Rhodococcus* sp. AD45 into the pGEM®T Easy vector (Promega, Madison, WI, USA) to be used as template DNA. The detection limit was 10^2^ copies per 20 μl reaction. Efficiency of all qPCRs ranged from 96–104%. *isoA* copies were normalised to 16S rRNA gene copy number (with the assumption of a rough ratio of 1:1 *isoA* to 16S rRNA gene within a genome) in order to estimate the relative abundance of isoprene degrading bacteria in each environmental sample.

Number of copies of 16S rRNA genes was determined by qPCR using 519F and 907R primers [75]. Reactions (20 μl) contained 10–70 pg DNA, 400 nM of each primer and 10 μl of SensiFast SYBR Hi-ROX kit. The qPCR reaction consisted of an initial denaturation step at 95 °C for 3 min, followed by 40 cycles of 95 °C for 20 s, 55 °C for 20 s and 72 °C for 30 s. Data were collected at 72 °C for 15 s. Specificity of the qPCR reaction and quantification of 16S rRNA gene copy number were determined as described above.

## Results and discussion

### Profiling the bacterial community associated with *Salix fragilis* leaves

To identify the active isoprene degraders associated with willow leaves, a DNA SIP experiment was set up using ^13^C-labelled isoprene in microcosms consisting of cells washed from leaves incubated in minimal medium with a headspace of isoprene vapour (150 ppmv). DNA was extracted from cells following consumption of approximately 0.5 µmol isoprene ml^−1^ (13–53 days). Density gradient ultracentrifugation and fractionation of DNA from samples incubated with ^12^C or ^13^C isoprene resulted in the recovery of light and heavy fractions from both sets of incubations, with heavy buoyant density samples from the ^13^C incubations containing the enriched isoprene degrading community. The DNA extracted from these samples was analysed by 16S rRNA gene amplicon sequencing and shotgun metagenomics.

Following denoising and removal of chimeric sequences, a total of 3,368 ASVs were obtained from 16S rRNA amplicon sequence data across all samples and replicates. The average number of cleaned and processed reads used to collate ASVs was 561,187.

**Fig. 1:**
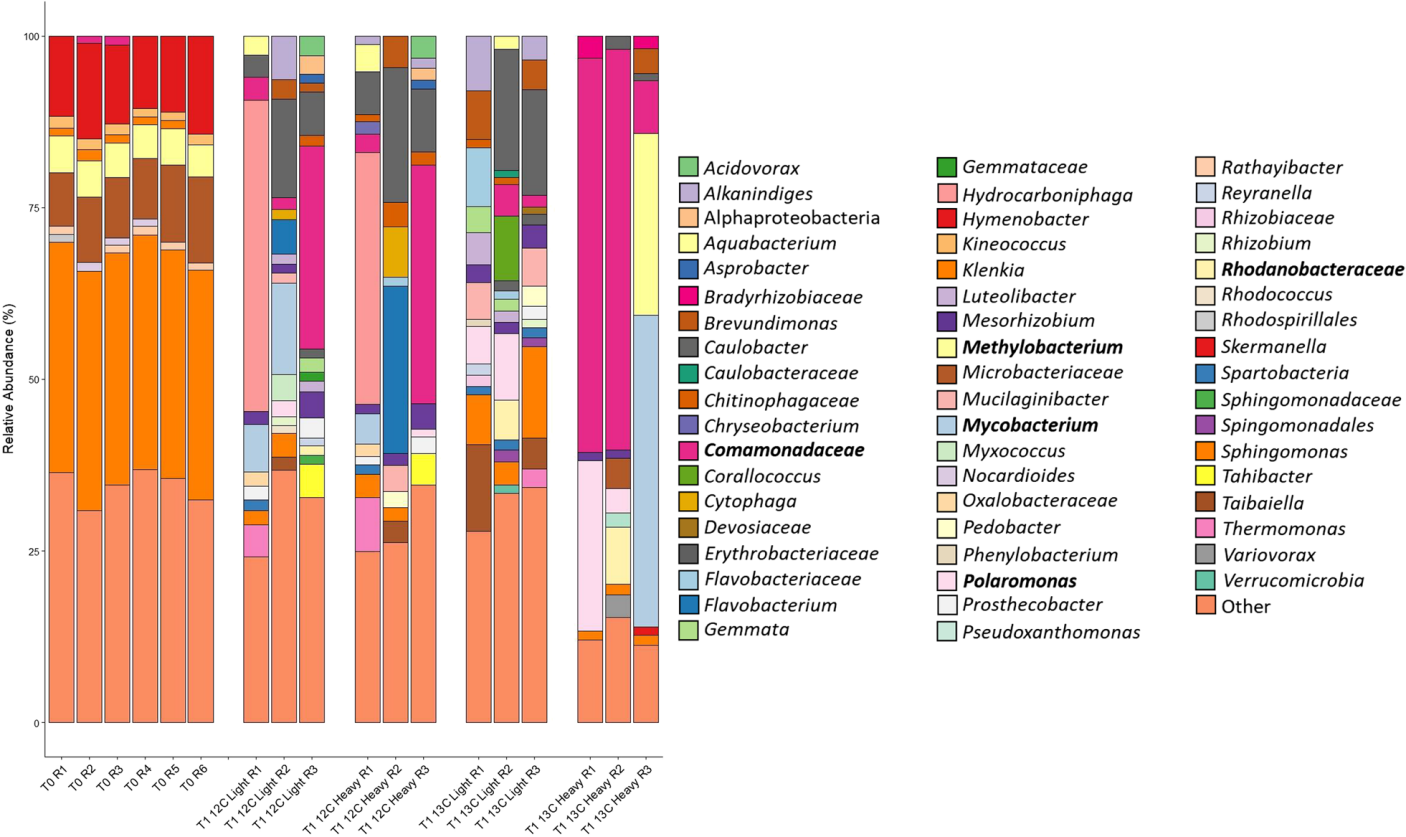
Bacterial community profile of DNA retrieved from willow leaf samples analysed by 16S rRNA gene amplicon sequencing. Samples are represented as unenriched (T0), enriched (T1), unlabelled (12C), labelled (13C), heavy DNA and light DNA fractions retrieved after DNA-SIP. R1–6 indicate the six replicate samples analysed. Taxa that are at less than 1% relative abundance in a sample are grouped as ‘Other’. Taxa that were of > 5% relative abundance in heavy fractions of ^13^C-enriched samples are in bold

In those samples incubated with isoprene, although there was a variation between replicates (Fig. 1), unlabelled samples (^12^C light and heavy, and ^13^C light) shared many similarities. The labelled, heavy fraction of ^13^C-incubated samples were clearly distinct from these controls, indicating that the enrichment of isoprene degrading bacteria was successful.

Unenriched T0 samples were consistent across all replicates and were dominated by *Sphingomonas* with an average relative abundance (RA) of 33.86 ± 0.57%. Other notable taxa present were *Microbacteriaceae* (RA of 9.78 ± 1.76%), *Hymenobacter* (RA of 12.19 ± 1.54%) and *Methylobacterium* (RA of 5.09 ± 0.26%).

Control light fraction samples incubated with ^12^C isoprene were quite distinct between replicates in terms of their bacterial diversity. Replicate 1 showed an abundance of *Hydrocarboniphaga* with an RA of 45.35% although the genus was not found in other replicates. *Mycobacterium* was found in replicates 1 and 2 with an RA of 6.94% and 13.32% respectively. *Comamonadaceae* was seen in all replicates with low RA in replicates 1 and 2 (RA of 3.39% and 1.75% respectively) but showed higher abundance in replicate 3, with an RA of 29.59%. *Caulobacter* was present in all replicates with an average RA of 7.91 ± 5.76%.

In practice, it is expected that DNA would be recovered from all fractions during the fractionation process, but in the heavy fractions of ^12^C-incubated samples, the only driver for a change in diversity would be caused by a particularly high GC content in a given taxa, this is the reason why such a low proportion of DNA is recovered from these samples (1% in this study). Outside of this occurrence, the heavy and light fractions of ^12^C-incubated samples were expected to be very similar, as can be seen in Fig. 1.

The light DNA fractions of samples incubated with isotopically labelled ^13^C isoprene contained the *Caulobacter* also seen in samples incubated with ^12^C, with an RA of 17.58% and 15.40% in replicates 2 and 3. Other than this, the light fractions of samples incubated with ^13^C do show some clear differences. *Sphingomonas* were present across all replicates with an average RA of 7.99 ± 5.06%, the presence of *Tabiella* in replicates 1 and 3 was also unique to this group of samples, with an RA of 12.69% and 4.46% respectively (Fig. 1).

Finally, the heavy fraction of samples incubated with ^13^C isoprene represent those bacteria that utilised the labelled isoprene during the process of reproduction and growth, confirming that these bacteria have the metabolic capacity to assimilate carbon from isoprene. The distinctive bacterial community seen in these samples indicates that the selective pressure introduced by the DNA SIP experiment was successful in enriching these bacterial taxa. A substantial increase in the relative abundance of *Comamonadaceae* was seen in replicates 1 and 2, with an RA of 57.48% and 58.33% respectively. *Polaromonas* was also enriched with an RA of 24.78% in replicate 1, although this was not present in the other two replicates. A very notable shift in the bacterial community was seen with replicate 3. Here the abundance of *Comamonadaceae* was not observed, and instead, there was a higher abundance of *Methylobacterium* (RA of 26.48%) which was not seen in any of the other enriched fractions or samples. However, as mentioned earlier, it was present in unenriched T0 samples (RA of 5.09 ± 0.26%). A substantial increase in abundance of *Mycobacterium* with an RA of 45.39% was also observed (Fig. 1).

**Fig. 2:**
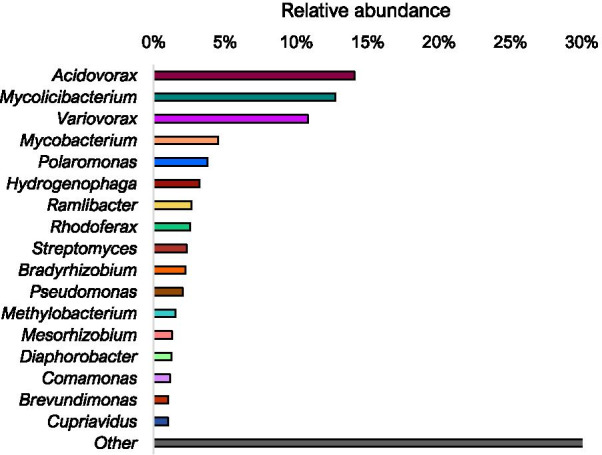
Relative abundance of bacterial taxa retrieved after analysis of the metagenome from pooled heavy fractions from ^13^C-labelled willow leaf samples retrieved after DNA-SIP. Metagenome data were analysed and classified taxonomically using Kraken [60]. All taxa that were at less than 1% relative abundance or could not be classified in the ^13^C-labelled DNA have been grouped as ‘Other’

Further analysis of the bacterial community structure was undertaken by examining metagenomic data obtained after DNA-SIP incubations as described in Materials and Methods. These data were analysed and taxonomically classified using Kraken [62] and revealed the presence of a number of genera belonging to the family *Comamonadaceae*, with *Acidovorax* (RA of 14%), *Variovorax* (RA of 10.83%), *Polaromonas* (RA of 3.8%)*, Hydrogenophaga* (RA of 3.2%)*, Ramlibacter* (RA of 2.7%) and *Rhodoferax* (RA of 2.5%) being recovered in ^13^C-labelled, heavy DNA (Fig. 2). The presence of a number of different genera of the *Comamonadacea* in this phyllosphere environment mirrors a previous study which focused on the bacterial community of soil associated with a willow species where *Comamonadaceae* made up 21–30% of the relative abundance in ^13^C-incubated heavy samples [42]. Although the two environments are different, this shared abundance might suggest members of the phyllosphere community are being transported to the bulk soil environment, possibly though rainfall or falling leaves.

*Bradyrhizobium* from the order *Rhizobiales* which was observed after 16S rRNA gene amplicon analysis (Fig. 1) was also observed after metagenome analyses with an RA of 2.23% (Fig. 2). *Mycolicibacterium* had been labelled with an RA of 12.72%. This genus, which has recently been differentiated from the genus *Mycobacterium* [76], was also found in the heavy DNA fractions arising from ^13^C-labelled SIP incubations after 16S rRNA gene amplicon analysis (Fig. 1). *Mycobacterium* itself made up 4.52% of the metagenomic community. Previously, *isoA* sequences sharing high sequence similarity with the *isoA* of *Mycobacterium* AT1 were estimated to make up half of all *isoA* sequences present on the leaves of sampled willow leaves [48]. However, this is the first instance of *Mycobacterium* being significantly enriched in ^13^C-incubated heavy fractions from a DNA SIP experiment with terrestrial samples.

*Methylobacterium*, which was estimated to be present at high relative abundance in 16S rRNA gene amplicon analysis of the heavy DNA fraction from ^13^C isoprene-incubated replicate 3, also featured in the metagenome analysis with an RA of 1.5%. The appearance of a ^13^C-labelled *Methylobacterium* here is interesting, since *Methylobacterium* species have previously been reported to grow on isoprene [77, 78] but are still quite rare in studies examining isoprene-degrading communities in the environment.

Without an extant example of the strains that make up the *Methylobacterium* ASVs, it cannot be said with absolute certainty that they do have the metabolic capability to degrade isoprene, but their presence in the heavy fraction of ^13^C isoprene-enriched samples (while not abundant in the heavy ^12^C controls) suggests they have indeed utilised the ^13^C-labelled isoprene during growth. However, there is the possibility that labelled by-products of isoprene metabolism produced by other organisms in the microcosm during isoprene degradation could have been assimilated by *Methylobacterium.* As the only methylotroph linked to isoprene degradation, further analysis confirming *Methylobacterium* to be an isoprene-degrading bacterium would be of great interest.

### Analysis of an abundant *Mycobacteriaceae* MAG containing two isoprene monooxygenase gene clusters

Contigs obtained from metagenomic data were binned and a number of metagenome assembled genomes (MAGs) were recovered (Table 1). Of these MAGs, one identified as belonging to the family *Mycobacteriaceae*, was selected for further investigation due to the presence of a complete isoprene (*iso*) metabolic gene cluster. This MAG was the most abundant of those recovered with 325 genome copies per million reads. On further investigation using the MiGA pipeline [74], the MAG was identified to genus level as *Mycobacterium.*

**Table 1:** Statistics for the completeness and abundance of recovered MAGs

Abundance Ranking	Completeness (%)	Contamination (%)	N50	Size (Mbp)	ID
1st	99.62	1.31	426,339	7.4	*Mycobacteriaceae*
2nd	84.53	2.30	28,949	4	*Comamonadaceae*
3rd	78.65	1.48	19,712	3.4	*Comamonadaceae*
4th	71.3	1.52	21,622	5	*Comamonadaceae*
5th	92.4	1.12	70,582	4.4	*Comamonadaceae*
6th	92.75	4.48	56,291	6.1	*Comamonadaceae*
7th	89.21	2.45	47,756	4	*Comamonadaceae*
8th	88.71	2.81	33,346	4.3	*Comamonadaceae*
9th	98.86	0.17	91,899	4.8	*Methylobacteriaceae*
10th	89.89	5.65	114,304	8.7	*Myxococcales*
11th	92.9	5.46	59,280	4.8	*Comamonadaceae*
12th	94.35	1.02	64,831	3.9	*Burkholderiales*
13th	98.77	0.80	142,624	4.2	*Xanthomonadaceae*
14th	77.75	8.04	36,155	3.9	*Comamonadaceae*
15th	98.98	0.76	201,989	3.3	*Microbacteriaceae*
16th	98.77	1.31	170,797	3.8	*Sphingomonadaceae*
17th	90.67	3.08	28,589	5.7	*Burkholderiales*
18th	87.71	0.53	197,524	2.8	*Caulobacteraceae*
19th	97.15	7.72	93,325	4.1	*Caulobacteraceae*
20th	95.4	1.74	263,888	4.2	*Bradyrhizobiaceae*
21st	91.19	13.04	33,095	3.2	*Caulobacteraceae*
22nd	96.75	1.15	93,753	3.4	*Xanthomonadaceae*
23rd	96.77	2.96	58,108	8.5	*Proteobacteria*
24th	93.05	2.47	50,463	8.2	NA
25th	88.3	3.19	14,127	3.5	*Alphaproteobacteria*
26th	88.11	0.93	49,319	3.8	NA
27th	91.77	0.69	14,215	3.3	*Nocardioidaceae*
28th	91.73	3.23	29,968	3.1	*Xanthomonadaceae*
29th	91.33	1.21	15,927	5.7	*Mycobacteriaceae*
30th	82.26	9.74	5,017	4.4	*Bradyrhizobiaceae*
31st	74.49	1.32	5,029	3.2	*Xanthomonadaceae*
32nd	73.68	1.85	3,730	2.5	*Alphaproteobacteria*
33rd	80.6	0.49	6,496	4.5	*Bacteroidetes*
34th	81.82	1.21	3,958	3.3	*Sphingobacteriaceae*

**Fig. 3:**
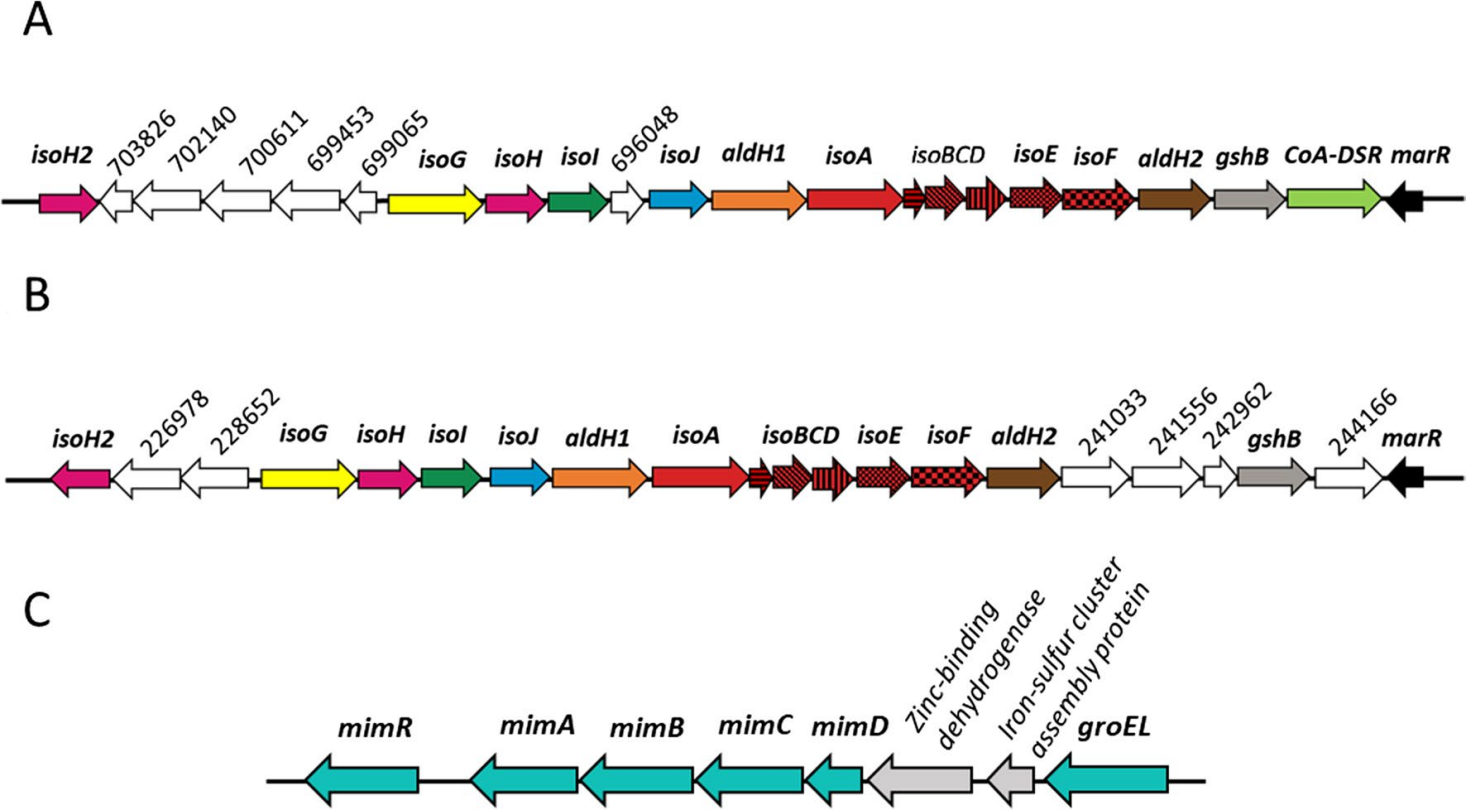
Isoprene degradation gene clusters recovered from MAGs assembled from heavy DNA incubated with ^13^C isoprene. **A**, **B** iso clusters 1 and 2 recovered from a *Mycobacterium* MAG assembled from heavy DNA retrieved after DNA-SIP. Genes encoding IsoMO (*isoABCDEF*) are coloured in red. Adjacent genes *isoGHIJ* and the duplicate gene *isoH2* encode a CoA transferase, dehydrogenase and two glutathione transferases involved in the subsequent steps of isoprene metabolism. Genes *aldH1, CoA-DSR, gshB* and *marR* encode an aldehyde dehydrogenase, a CoA-disulfide reductase, a glutathione synthase and a putative transcriptional regulator respectively. Adjacent genes that are not yet known to be involved in isoprene degradation are coloured in white. (696 048—Hypothetical protein; 699 065—Hypothetical protein; 699 453—Hypothetical protein; 700 611—Triacylglycerol lipase; 702 140—Acetyl-CoA-acetyltransferase; 703 826—AraC family transcriptional regulator; 226 978—Acetyl-CoA-acetyltransferase; 228 652—Hypothetical protein; 241 033—Hypothetical protein; 241 556—Hypothetical protein; 242 962—CaiB/BaiF family protein; 244 166—FAD-dependant oxidoreductase). Regulatory genes are shown in black. **C** A propane monooxygenase gene cluster recovered from a *Mycobacterium* MAG**.** Genes associated with propane metabolism are coloured in blue. Genes *mimABCD* encode an oxygenase large subunit, a reductase, an oxygenase small unit and a coupling protein respectively, making up the propane monooxygenase, with *groEL* encoding an associated chaperonin [75, 76]. Adjacent genes not involved in propane metabolism are coloured in grey

The *Mycobacterium* MAG has two non-identical copies of the *iso* gene cluster *isoABCDEFGHIJ* (referred to as *iso* cluster 1 and *iso* cluster 2), encoding enzymes of the isoprene degradation pathway (reviewed in [41, 43]), together with associated genes *aldH1, CoA-DSR, gshB* and *marR,* encoding an aldehyde dehydrogenase, a CoA-disulfide reductase, a glutathione synthase and a putative transcriptional regulator respectively (Fig. 3A, B).

To rule out the possibility that one of these duplicate clusters was an artefact of assembly or contamination, the contigs containing both clusters were investigated for the presence of essential marker genes, and each marker gene was analysed for possible duplication. Both contigs were of substantial length (995,005 bp and 363,049 bp) with coverage > 400× , and contained single copies of marker genes consistent with the genome of a member of the *Mycobacteriaceae*, which strongly suggested that there was no contamination and that this MAG did indeed contain two *iso* gene clusters. This *Mycobacterium* MAG was also investigated for genes that would indicate the presence of a plasmid but none were found, suggesting that both *iso* gene clusters are located on the genome and are not plasmid-borne as found in *Rhodococcus* strain AD45 and *Variovorax* strain WS11 [37, 79], two isoprene degrading strains that contain duplicated genes within a single isoprene degradation cluster, but do not contain duplicate copies of the full *iso* gene cluster.

The translated polypeptide sequences of *iso* genes from both clusters showed a high degree of identity (> 75% amino acid identity) to the corresponding polypeptides found in the isoprene-degrading *Mycobacterium* AT1 [36], although *Mycobacterium* AT1 also contained only one copy of the *iso* metabolic gene cluster (Additional file 1: Table S1).

### Identification of a propane monooxygenase gene cluster in *Mycobacterium*

The genome of the recovered *Mycobacterium* MAG was investigated for other metabolic genes of interest and a full propane monooxygenase gene cluster was recovered which comprised of genes *mimABCD* and the propane monooxygenase operon transcriptional regulator *mimR,* along with the associated chaperonin *groEL* (Fig. 3C) [80]. The propane monooxygenase is a binuclear iron monooxygenase encoded by genes *mimABCD* that encode an oxygenase large subunit, a reductase, an oxygenase small unit and a coupling protein respectively. Such gene clusters share high amino acid identity to the propane monooxygenase encoded by the *prmABCD* gene clusters found in *Rhodococcus* sp. strain RHA1 [81] and *Gordonia* sp. strain TY‐5 [82]. The propane monooxygenase in these bacteria is essential for propane and acetone metabolism and is capable of oxidizing phenol to hydroquinone in the presence of acetone [83]. However, the propane monooxygenase found in *Mycobacterium* AT1 did not allow growth on phenol [36].

The recovered MimABCDR polypeptides all shared a high amino acid identity (> 97%) with the corresponding polypeptides found in *Mycobacterium* AT1 (Additional file 1: Table S2), a bacterium that could grow on propane and ethane [36, 84]. However, amino acid identity (AAI) analysis of the genome as a whole gave a shared identity of 92.09%, indicating that although they are closely related, they are not the same species (same species share an AAI of 95% or above). Additional file 1: Table S2 also shows comparison of Mim polypeptides to the well-characterised *Mycobacterium smegmatis* strain mc^2^155 in which, alongside propane and acetone metabolism, *mimABCD* encode enzymes responsible for the regioselective oxidation of phenol to hydroquinone, similar to those of *Rhodococcus* sp. strain RHA1 and the *Gordonia* sp. strain TY‐5 mentioned earlier [80, 83].

### Recovery of a *Methylobacterium* MAG, present in the isoprene-degrading community as revealed by DNA-SIP

Another MAG, identified as *Methylobacteriaceae*, showed high (98.86%) completion and low (0.17%) contamination (Table 1). It was also one of the most abundant MAGs recovered from metagenome data, following *Comamonadaceae* and *Mycobacterium,* with 49 genome copies per million reads*. Methylobacterium*, a member of the *Methylobacteriaceae* family, were found to be notably enriched in the heavy fraction of one of the ^13^C-incubated samples in 16S rRNA analysis (Fig. 1), however this MAG contained no *iso* genes or obvious alternatives that might provide the microbe with the metabolic ability to degrade isoprene.

Further analysis recovered a full *mxa* methylotrophy gene cluster encoding a calcium-containing methanol dehydrogenase (*mxaFJGIRSACKLDEHB*) with an upstream *mxaW,* a methanol regulated gene of unknown function [85, 86]. In addition, six genes required for pyrroloquinoline quinone (PQQ) synthesis were found, (*pqqABC/DE*) and (*pqqFG*)[86]. Genes *mxbDM* and *mxcQE* involved in transcriptional regulation of the methanol oxidation system were also present. Comparison between these genes and the same clusters from the well characterised *Methylobacterium extorquens* AM1 can be seen in Additional file 1: Table S3 [87].

### Abundance of bacteria associated with the leaves of willow and other plant species encoding for the isoprene monooxygenase alpha-subunit

**Fig. 4:**
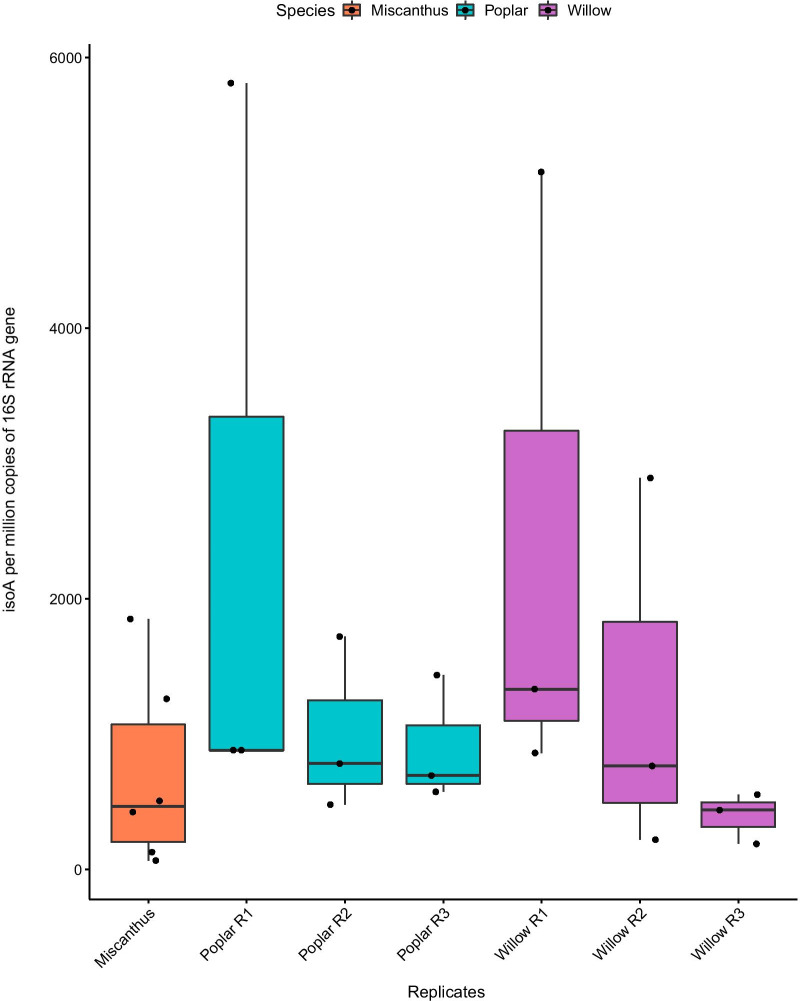
Relative abundance of isoprene degraders in DNA retrieved from samples of leaf washings from Miscanthus, poplar and willow trees. Samples were analysed by qPCR of *isoA* genes (48). *isoA* copies were normalised to the number of 16S rRNA gene copies present in each DNA sample. The three replicates for poplar and willow samples represent individual trees, while data points within replicates each represent 2 g of leaves taken from around each tree. Data points with samples from *Miscanthus* each represent a single grass leaf

The DNA-SIP experiments described earlier enabled the identification of active isoprene-degrading bacteria on the surface of willow leaves. Analysis of MAGs retrieved from heavy DNA after DNA-SIP experiments also confirmed that isoprene-degraders present contained *iso* metabolic gene clusters with significant identity to those of well-characterised isoprene-degraders and that *isoA*, encoding the putative active site of IsoMO was again highly conserved in these MAGs. In order to gain initial insights into the relative abundance of isoprene degraders on willow leaves and to compare with other tree species, the abundance of *isoA*-containing bacteria associated with leaves of the willow bioenergy crop *Salix viminalis,* was investigated with the use of qPCR following methods described previously [48]. For comparison, the leaves of the high isoprene-producing crop *Poplar nigra* and the non-producer *Miscanthus* x *giganteus* were also analysed. Results showed that the willow leaves sampled harboured an average of 1379 ± 1030 *isoA* sequences per million copies of 16S rRNA genes, indicating an average of ~ 0.14%. In comparison, leaves of the high isoprene-emitting poplar species contained 1473 ± 911 *isoA* sequences per million 16S rRNA genes, equating to approximately 0.15%. The non-emitting *Miscanthus* species showed 801 ± 704 *isoA* sequences per million 16S rRNA genes, representing about 0.07% (Fig. 4). While the average number of 16S rRNA genes per species can vary widely between taxa and it cannot be assumed that *isoA* genes are present in bacterial genomes in a 1:1 ratio with 16S rRNA genes, this metric can only act as an approximation of the abundance of isoprene degrading bacteria in the wider bacterial community. As such, these results demonstrate a much higher abundance of *isoA*-containing bacteria in isoprene-rich leaf environments compared to leaves of a plant that does not emit isoprene. However, in comparisons between species that emit isoprene, there does not appear to be a linear correlation between isoprene production and number of isoprene degrading bacteria. It has been reported that *Populus nigra* emits 29–76 μg g^−1^ (dry weight) h^−1^ of isoprene, while *Salix viminalis* emits 80–130 μg g^−1^ (dry weight) h^−1^ [49, 88–90]. Estimated numbers of potential isoprene degrading bacteria found in willow samples however were marginally lower than those in poplar samples. This preliminary examination of the abundance of *isoA* sequences associated with isoprene-degrading trees confirms at least their presence and relatively high abundance when compared to a non-isoprene producing crop plant (*Miscanthus*). However, these data need to be interpreted with caution since the qPCR data were highly variable between plants and this may have been due to the ease (or otherwise) with which bacteria can be removed from different types of leaves. Our DNA-SIP study and *isoA* assays provide proof-of principle for the study of isoprene cycling in the environment and clearly show that isoprene-degrading bacteria are present in significant numbers in these environments but in future, a more systematic, quantitative study of isoprene-degrading bacteria, comparing a wide variety of high- and low-isoprene emitting trees, will be required. This could include analysis of transcripts to look more closely at the difference in isoprene degrading activity between such species.

## Conclusions

Focussed metagenomics using DNA-SIP with ^13^C-labelled isoprene and leaf washings from a high isoprene-emitting willow tree enabled identification of active isoprene-degrading bacteria from this environment. Active isoprene-degraders included various members of the *Comamonadaceae* family and the Actinobacteria phylum. Analysis of metagenome sequence data from heavy DNA retrieved after SIP experiments enabled the assembly of a MAG from a putative-isoprene degrading *Mycobacterium* which contained at least two soluble diiron-containing monooxygenase gene clusters; duplicate copies of the *iso* metabolic cluster (*isoABCDEFGHIJ*), together with a putative propane monooxygenase gene cluster (*mimABCD*). Also of particular interest was a putative isoprene degrading *Methylobacterium* which warrants further study. These cultivation-independent approaches provide DNA sequence data to assist targeted isolation of isoprene degrading bacteria from the phyllosphere and provide proof-of-concept for more detailed quantitative studies on isoprene-degraders present on the leaves of high-isoprene-emitting trees.

## Supplementary Information

**Additional file 1:Table S1.** Comparison of polypeptides recovered from the duplicate isoprene degradation gene clusters (*iso* cluster 1 and *iso* cluster 2; Figure 3) found in a *Mycobacterium* MAG to those recovered from *Mycobacterium* AT1 and the well-characterised *Rhodococcus* AD45. **Table S2.** Comparison of polypeptides recovered from a propane monooxygenase gene cluster recovered from a *Mycobacterium* MAG to those recovered from *Mycobacterium* AT1 and the well-characterised *Mycobacterium smegmatis m*^*c*^*2155*. **Table S3.** Comparison of polypeptides associated with the oxidation of methanol to formaldehyde recovered from a *Methylobacteriaceae* MAG, compared to the well characterised *Methylobacterium extorquens* AM1. **Fig. S1.** Percentage of DNA retrieved as a function of the density of each fraction following density gradient ultracentrifugation.

## Data Availability

Amplicon sequencing and metagenomic reads are available from the sequence read archive (SRA) under Bioproject PRJNA272922 (amplicon Biosamples SAMN18058298—SAMN18058333, metagenomic Biosamples SAMN18095267—SAMN18095269).
