# Supplementary material for: Isoprene-degrading bacteria associated with the phyllosphere of Salix fragilis, a high isoprene-emitting willow of the Northern Hemisphere
Source: Environ Microbiome. 2021 Aug 26;16:17. doi: 10.1186/s40793-021-00386-x (PMC8394569; doi:10.1186/s40793-021-00386-x)
Supplement: Supplementary file 1 — Additional file 1:Table S1. Comparison of polypeptides recovered from the duplicate isoprene degradation gene clusters (iso cluster 1 and iso cluster 2; Figure 3) found in a Mycobacterium MAG to those recovered from Mycobacterium AT1 and the well-characterised Rhodococcus AD45. Table S2. Comparison of polypeptides recovered from a propane monooxygenase gene cluster recovered from a Mycobacterium MAG to those recovered from Mycobacterium AT1 and the well-characterised Mycobacterium smegmatis mc2155. Table S3. Comparison of polypeptides associated with the oxidation of methanol to formaldehyde recovered from a Methylobacteriaceae MAG, compared to the well characterised Methylobacterium extorquens AM1. Fig. S1. Percentage of DNA retrieved as a function of the density of each fraction following density gradient ultracentrifugation. [file 40793_2021_386_MOESM1_ESM.docx]

Supplementary information for

**Isoprene-degrading bacteria associated with the phyllosphere of *Salix fragilis,* a high isoprene-emitting willow of the Northern Hemisphere.**

Lisa Gibson^1^, Andrew T. Crombie^2^, Niall P. McNamara^3^, J. Colin Murrell^1*^

^1^School of Environmental Sciences, University of East Anglia, Norwich Research Park, Norwich, NR4 7TJ, UK

^2^School of Biological Sciences, University of East Anglia, Norwich Research Park, Norwich, NR4 7TJ, UK

^3^Centre of Ecology and Hydrology, Lancaster University, Bailrigg, Lancaster, LA1 4AP, UK

*Corresponding authors:

Lisa Gibson, ^1^School of Environmental Sciences, University of East Anglia

Norwich Research Park, NR4 7TJ, UK

E-mail: lisa.gibson@uea.ac.uk

J Colin Murrell, ^1^School of Environmental Sciences, University of East Anglia

Norwich Research Park, NR4 7TJ, UK

E-mail: j.c.murrell@uea.ac.uk

**Table S1. Comparison of polypeptides recovered from the duplicate isoprene degradation gene clusters (*iso* cluster 1 and *iso* cluster 2; Figure 3) found in a *Mycobacterium* MAG to those recovered from *Mycobacterium* AT1 and the well-characterised *Rhodococcus* AD45** [1, 2]**.**

|  |  | **Comparison to *Mycobacterium* AT1** | | **Comparison to *Rhodococcus* AD45** | |
| --- | --- | --- | --- | --- | --- |
| **Polypeptide** | **Cluster** | **Coverage** | **ID (aa%)** | **Coverage** | **ID (aa%)** |
| **IsoA** | *1* | 100 | 91 | 100 | 86.69 |
|  | *2* | 96 | 96.8 | 100 | 82.24 |
| **IsoB** | *1* | 98 | 79.57 | 100 | 69.15 |
|  | *2* | 100 | 85.1 | 98 | 70.97 |
| **IsoC** | *1* | 95 | 89.8 | 92 | 75.24 |
|  | *2* | 100 | 90.4 | 99 | 72.57 |
| **IsoD** | *1* | 98 | 92.2 | 100 | 81.9 |
|  | *2* | 98 | 96.3 | 100 | 79.09 |
| **IsoE** | *1* | 100 | 83.6 | 98 | 75.67 |
|  | *2* | 100 | 85.7 | 99 | 76.11 |
| **IsoF** | *1* | 97 | 79.5 | 98 | 63.53 |
|  | *2* | 97 | 80.9 | 97 | 63.45 |
| **IsoG** | *1* | 99 | 91.8 | 100 | 85.43 |
|  | *2* | 100 | 93.3 | 100 | 83.29 |
| **IsoH** | *1* | 100 | 89.8 | 100 | 78.76 |
|  | *2* | 100 | 92 | 100 | 79.2 |
| **IsoI** | *1* | 100 | 93.7 | 100 | 81.51 |
|  | *2* | 100 | 94.1 | 100 | 85.29 |
| **IsoJ** | *1* | 99 | 88.4 | 99 | 75.86 |
|  | *2* | 99 | 89.3 | 99 | 75.86 |

Figures are given as a percentage of shared amino acid (aa) identity (ID). IsoABCDEF make up the isoprene monooxygenase IsoMO, responsible for the first step of the isoprene degradation pathway. IsoGHIJ, (a CoA transferase, dehydrogenase and two glutathione transferases) comprise the subsequent steps of isoprene metabolism.

|  | **Comparison to**  ***Mycobacterium* AT1** | | **Comparison to**  ***Mycobacterium smegmatis*** | |
| --- | --- | --- | --- | --- |
| **Polypeptide** | **Coverage** | **ID (aa%)** | **Coverage** | **ID (aa%)** |
| **MimR** | 100 | 97.78 | 99 | 77.87 |
| **MimA** | 99 | 98.7 | 100 | 97.23 |
| **MimB** | 100 | 98.56 | 100 | 88.79 |
| **MimC** | 100 | 99.46 | 98 | 91.3 |
| **MimD** | 100 | 96.61 | 94 | 93.69 |
| **GroEL** | 100 | 99.09 | 98 | 49.72 |

**Table S2. Comparison of polypeptides recovered from a propane monooxygenase gene cluster recovered from a *Mycobacterium* MAG to those recovered from *Mycobacterium* AT1 and the well-characterised *Mycobacterium smegmatis m^c^2155*** [3]**.**

Figures are given as a percentage of shared amino acid (aa) identity (ID). MimABCD, an oxygenase large subunit, a reductase, an oxygenase small unit and a coupling protein respectively, make up the propane monooxygenase, with GroEL acting as an associated chaperonin [4, 5].

**Table S3.** **Comparison of polypeptides associated with the oxidation of methanol to formaldehyde recovered from a *Methylobacteriaceae* MAG, compared to the well characterised *Methylobacterium extorquens* AM1** [6]

|  | **Comparison to *Methylobacterium extorquens* AM1** | |
| --- | --- | --- |
| **Polypeptide** | **Coverage** | **ID (aa%)** |
| **MxaF** | 100 | 95.37 |
| **MxaJ** | 100 | 84.19 |
| **MxaG** | 100 | 89.34 |
| **MxaI** | 100 | 95.83 |
| **MxaR** | 100 | 90.96 |
| **MxaS** | 97 | 81.29 |
| **MxaA** | 83 | 65.44 |
| **MxaC** | 100 | 82 |
| **MxaK** | 96 | 72.2 |
| **MxaL** | 93 | 80 |
| **MxaD** | 96 | 76.88 |
| **MxaE** | 98 | 68.08 |
| **MxaH** | 93 | 72.28 |
| **MxaB** | 93 | 72.28 |
| **MxaW** | 79 | 68.4 |
| **PqqA** | 100 | 96.55 |
| **PqqB** | 100 | 81.61 |
| **PqqC/D** | 100 | 78.42 |
| **PqqE** | 100 | 86.86 |
| **PqqF** | 98 | 84.55 |
| **PqqG** | 98 | 78.30 |
| **MxbD** | 97 | 70.17 |
| **MxbM** | 99 | 85.84 |
| **MxcQ** | 93 | 66.31 |
| **MxcE** | 90 | 87.09 |

Figures are given as a percentage of shared amino acid (aa) identity (ID). Mxa polypeptides are involved in C1 metabolism in *M. extorquens* and PQQ polypeptides are involved in the synthesis of pyrolloquinoline quinone (PQQ) a cofactor of methanol dehydrogenase. The designations used are as described in [7].


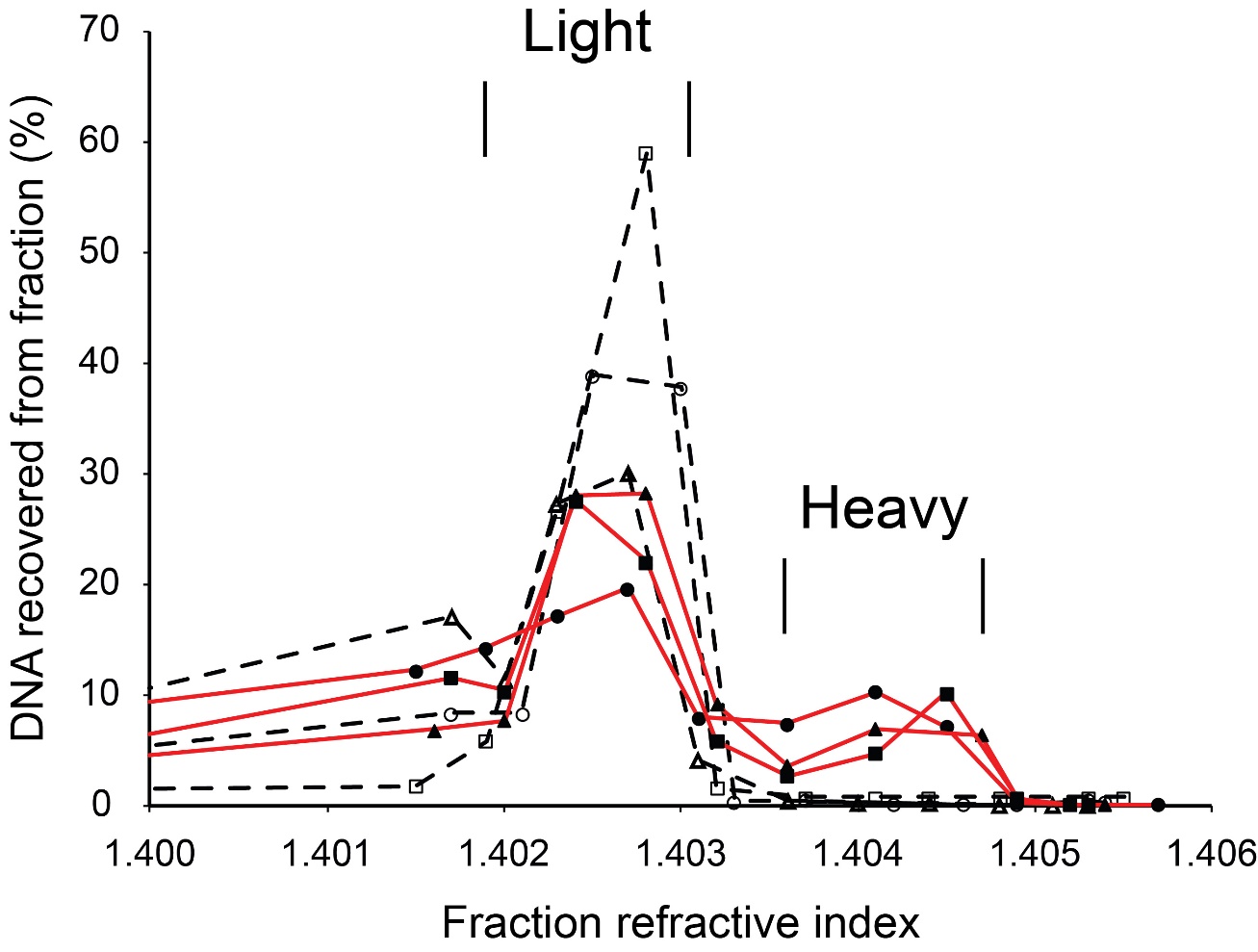


**Fig. S1.** **Percentage of DNA retrieved as a function of the density of each fraction following density gradient ultracentrifugation.** Black dotted lines represent samples incubated with ^12^C isoprene. Solid red lines represent samples incubated with ^13^C isoprene. The refractive index was determined for individual fractions recovered from CsCl gradients.

**References**

1. van Hylckama Vlieg JE, Kingma J, van den Wijngaard AJ, Janssen DB. A glutathione S-transferase with activity towards cis-1, 2-dichloroepoxyethane is involved in isoprene utilization by *Rhodococcus* sp. strain AD45. Appl Environ Microbiol. 1998;64:2800–5.

2. Johnston A, Crombie AT, Khawand M El, Sims L, Whited GM, McGenity TJ, et al. Identification and characterisation of isoprene-degrading bacteria in an estuarine environment. Environ Microbiol. 2017;19:3526–37.

3. Snapper SB, Melton RE, Mustafa S, Kieser T, Jr WRJ. Isolation and characterization of efficient plasmid transformation mutants of *Mycobacterium* *smegmatis*. Mol Microbiol. 1990;4:1911–9.

4. Sharp JO, Sales CM, LeBlanc JC, Liu J, Wood TK, Eltis LD, et al. An inducible propane monooxygenase is responsible for N- nitrosodimethylamine degradation by *Rhodococcus* sp. strain RHA1. Appl Environ Microbiol. 2007;73:6930–8.

5. Kotani T, Yamamoto T, Yurimoto H, Sakai Y, Kato N. Propane monooxygenase and NAD+-dependent secondary alcohol dehydrogenase in propane metabolism by *Gordonia* sp. strain TY-5. J Bacteriol. 2003;185:7120–8.

6. Anthony C. The Biochemistry of Methylotrophs.

7. Zhang M, Lidstrom ME. Promoters and transcripts for genes involved in methanol oxidation in *Methylobacterium* *extorquens* AM1. Microbiology. 2003;149:1033–40.
